# Social Prescribing in National Health Service Primary Care: What Are the Ethical Considerations?

**DOI:** 10.1111/1468-0009.12516

**Published:** 2021-06-25

**Authors:** REBECCA C.H. BROWN, KAMAL MAHTANI, AMADEA TURK, STEPHANIE TIERNEY

**Affiliations:** ^1^ Oxford Uehiro Centre for Practical Ethics University of Oxford; ^2^ Centre for Evidence Based Medicine Nuffield Department of Primary Care Health Sciences University of Oxford

## Abstract

**Policy Points**

- Social prescribing is proposed as a way of improving patients’ health and well‐being by attending to their non‐clinical needs. This is done by connecting patients with community assets (typically voluntary or charitable organizations) that provide social and personal support.
- In the United Kingdom, social prescribing is used to improve patient well‐being and reduce use of National Health Service resources.
- Although social prescribing schemes hold promise, evidence of their effects and effectiveness is sparse.
- As more information on social prescribing is gathered, it will be important to consider the associated ethical issues for patients, clinicians, link workers, and community assets.

Social prescribing is an effort to recognize and address the broader, nonclinical contributors to health and well‐being made by social and economic factors, while maintaining the clinical role of medically trained staff. It offers support to patients who have unmet social and personal needs (e.g., loneliness, debt, insecure housing, and bereavement) that can adversely impact on their health^1^ by directing them toward locally available nonclinical services such as support groups and activity schemes. Social prescribing may facilitate greater patient activation, including improved self‐management of long‐term conditions, by building connections and networks around individuals, meaning they are less dependent on health professionals for support.^2^ See Box 1 for a glossary of terms related to social prescribing.

**Box 1. Glossary of Terms Related to Social Prescribing and the UK Health Care System**

**Clinical care**: Interventions by physicians and other medically trained personnel that are largely medical in nature.

**Community assets**: A **range** of resources that strengthen a local community's capacity to develop and support health and well‐being initiatives, including physical infrastructure (buildings and other facilities), knowledge, and support networks.

**General practitioner (GP)**: The job title for a primary care physician in the United Kingdom; GPs are often (though not always) responsible for referring patients to social prescribing services.

**Link worker**: A specific role created to facilitate social prescribing by liaising between GPs, voluntary and community services, and patients. The role is varied and can incorporate different responsibilities. Also referred to as **community link worker**, **care coordinator**, or **care navigator**.

**National Health Service (NHS)**: The **nationalized** health care provider in the United Kingdom.

**Nonclinical care**: Nonmedical patient interventions to recognize and address contributors to health and well‐being made by social and **economic** factors.

**Primary care network**: A geographically based partnership of GP practices, typically covering populations of 30,000‐50,000 patients.

**Social prescribing**: A tool for supporting people's health and well‐being by directing them to nonclinical services (such as walking groups, befriending services, or cultural activities) in their local community. Also known as **community referral**.

**Voluntary and community sector (VCS)**: Organizations such as charities used to deliver social prescribing through, for instance, support and **activity** groups.

In the United Kingdom, social prescribing has become a part of mainstream National Health Service (NHS) service provision. The NHS Long Term Plan pledged to recruit 1,000 link workers by 2020/2021 as part of newly formed primary care networks,^3^ and since the COVID‐19 pandemic began, additional funds have been made available to support this recruitment drive.^4^ The NHS employs link workers to meet with patients for an extended time period, discuss their needs, help them set goals and develop an action plan, and direct them toward available services (typically voluntary and community organizations). Patients are referred to link workers via general practitioners (GPs) and other members of their primary care team, including nurses, midwives, and receptionists. It is also possible for people to self‐refer for some services.

Social prescribing is growing internationally, with initiatives in Canada, the United States, Australia, New Zealand,^5^, ^6^, ^7^, ^8^, ^9^ and elsewhere. At present, however, the United Kingdom seems to be leading the way in establishing formal, national social prescribing pathways, with explicit commitments to these activities through, for example, the planned recruitment of link workers outlined in the NHS Long Term Plan.^3^

The term “social prescribing” is not used in every country, and the formal and informal structures used to deliver this kind of holistic care may differ across countries and regions. In discussing social prescribing in the United Kingdom and the United States, Alderwick and colleagues^5^ noted that “approaches vary, but the process usually involves screening for social needs (such as social isolation or access to food), referring to community‐based services (such as welfare advice or housing support), and supporting people to access relevant services (often using a care coordinator or link worker).” Factors that shape how social prescribing operates at a fine‐grained level include existing social and health infrastructures in specific countries/localities, community needs, and relevant drivers of health and well‐being. For instance, social workers, occupational therapists, and a range of other allied health and social service professionals deliver types of social prescribing services.

In the United Kingdom, approximately one‐fifth of consultations are spent managing nonclinical problems,^10^ and part of the rationale for social prescribing and the promotion of link workers is to reduce use of NHS services while improving patient well‐being.^11^ At present, the policy goal for social prescribing is ambitious, with supporting evidence of its value still emerging. Some research suggests social prescribing could have benefits,^12^, ^13^ but robust evidence is sparse.^14^, ^15^ More research is required that explores the variety of ways social prescribing is practiced, and the successes and failures of different approaches that are employed.

As part of this descriptive and evaluative research, it is essential to evaluate the normative dimensions of social prescribing, and to look at potential ethical concerns, alongside studying its implementation and effectiveness. This paper outlines the kinds of considerations that will be necessary for an overall assessment of the ethical use and value of social prescribing as it becomes more widely adopted within mainstream health care.

## Why Ethics Matters

The introduction of any new intervention within health care attracts obvious questions relating to effectiveness. These questions are important, but it is also important to consider how activities aiming to promote health and well‐being relate to other things that matter. We offer an overview of some ways in which social prescribing interacts with ethical questions. We structure this discussion around three key groups involved in social prescribing: health care providers, patients, and the voluntary‐community sector (VCS). This roughly mirrors the NHS common outcomes framework, which judges “what good social prescribing looks like” in relation to its impact on the person, community groups, and the health and care system.^16^

Although social prescribing is not entirely new in its current form or as a function of general practice, it has features that set it apart from practices typically discussed by medical ethicists. In particular, social prescribing differs in its use of the VCS and often lacks a specific target or aim (beyond an aspiration to provide holistic support for nonclinical needs and to contribute to health and well‐being generally). There is a great deal of variability in how social prescribing is actually done.^17^ This may turn out to facilitate valuable flexibility, or lead to inconsistency. Such variability in what social prescribing schemes involve makes evaluation of their effectiveness unwieldy.

The current discussion will be necessarily speculative and open ended for multiple reasons. First, attempts to understand the ethics of social prescribing can—and often will—be informed by empirical evidence about the variety of ways in which social prescribing operates and the impact it engenders. As such, the current lack of robust evidence regarding the impact of social prescribing limits the breadth and confidence of an ethical analysis. Second, the diversity in how social prescribing services are designed and delivered, both within the United Kingdom and elsewhere, adds to the complexity of discussing social prescribing in general and evaluation of its ethical and practical implications. Third, there is no clear demarcation between problems, and ways of addressing those problems, that are clinical in nature and those that are not. Although we use this distinction and hope that it is helpful for the following discussion, we recognise that there will be many borderline cases.

### Social Prescribing and Patients

#### Patient Experience

It is important to understand the effect that social prescribing has on patients’ service use, health‐related outcomes, and broader components of well‐being. Several qualitative studies provide some evidence regarding patients’ experiences of social prescribing.^14^, ^18^, ^19^, ^20^ Patients might regard (and welcome) social prescribing as a genuine attempt to expand the way care is offered and the forms it takes as a complement to existing health care provisions. From this perspective, social prescribing directs patients toward services that are best able to meet their needs, thereby increasing patient satisfaction and well‐being by recognizing the difficulty of their situations and the kinds of support likely to be of value.^21^, ^22^, ^23^

It cannot, however, be assumed that improved patient experiences will be the case invariably or typically. If social prescribing is simply employed to relieve pressure on NHS (or other health care) services, particularly in general practice, there is a risk of it being used inappropriately to achieve this end, at the expense of good patient care. For instance, if alternative resources are lacking, GPs might “off‐load” to social prescribing services those patients who are regarded as “difficult” or who require more intensive support than social prescribing is designed to provide.^22^, ^24^

Even if the referring practitioner thinks social prescribing is appropriate, it might not be embraced fully by patients. Some patients may value the social legitimacy that attaches to receiving physician attention, which makes them feel that their condition and struggles are being taken seriously. A physician's care may also convey the legitimacy of the patient's health concerns to other individuals (e.g., friends, relatives, employers), and this legitimacy could be undermined if, instead, the patient is directed toward social prescribing services.^25^ Whether the value of social legitimation of one's suffering should be considered an appropriate reason for providing particular kinds of medical care is a question that needs addressing. It is worth noting that other kinds of non‐health‐related consequences of medical support, such as reassurance, might routinely be accepted as worthwhile benefits of health care.^26^, ^27^

#### Social Justice

We should consider the impact of social prescribing on existing social inequalities. The “inverse care law” posits that those most in need of care are least likely to receive it, and vice versa.^28^ Almost by definition, the patients for whom social prescribing is imagined to be of greatest benefit are precisely those hard‐to‐reach groups who are less likely to engage (such as people who are socially isolated and struggling to establish supportive social relationships).

A further concerning feature is that the reliance of social prescribing on charities and community groups means that areas where there are few such organizations might not be able to support social prescribing effectively. This risk is exacerbated by the differential effect of, for example, austerity cuts on UK public sector services which have disproportionately affected areas of higher social deprivation.^29^, ^30^ The 2008 economic crash, subsequent recession, and austerity policies have also led to a “hollowing out” of the UK charitable sector.^31^ Similarly, in the United States, a lack of available community resources has been identified as a threat to initiatives to support people's nonclinical needs.^5^

Therefore, even if social prescribing is effective for some, it may fail to help those most in need, and it could exacerbate existing inequalities unless it is delivered in a thoughtful manner, with inequality monitored and mitigated. There is a general presumption, among academics and laypeople, that inequality matters.^32^ However, questions about precisely what kinds of inequality are relevant and how much inequality is acceptable are far from settled. At the least, we should be concerned about interventions that seem to actively contribute to inequality, and consider whether the funds for such programs could be effectively used in ways that reduce inequality.

Social prescribing can encourage individual patients to become more actively engaged (via link workers, community programs, lifestyle changes, etc) with their health and specific nonclinical needs. However, this individualized approach may rely on the kinds of personal, social, and economic resources that those who could benefit from social prescribing may be lacking. Addressing the nonclinical needs of people on the wrong end of social inequality may require broader structural changes. If this is the case, social prescribing, even if modestly effective, could fail to make significant contributions to the lives and well‐being of those people for whom it is intended. In this case, social prescribing risks being a distraction that allows policymakers to give the *appearance* of addressing health inequalities without taking actions that would more effectively fulfill people's broader social and economic needs. More effective actions could be extremely demanding—for instance, addressing upstream factors such as poverty—and so it may be appealing to adopt strategies like social prescribing that are cheaper and less disruptive, but may ultimately be of limited (sustainable) value.

#### Service Delivery

One factor that could hinder people's capacity to benefit from social prescribing is the time‐limited nature of their contact with link workers. The NHS often restricts contact to a set number of sessions, though restrictions can vary from service to service. If patients lack the social support networks that typically contribute to a good and fulfilling life, their experience of isolation may be exacerbated by rationed contact with link workers. If relationship building and continuity of care are found to be important features of successful social prescribing schemes,^2^ it will be necessary to ensure that link workers have sufficient capacity to spend time with patients, when required, to achieve these goals—a potentially costly commitment.

#### Nomenclature

Another noteworthy ethical consideration relating to patients and social prescribing schemes concerns language. When we have participated in public engagement events,^33^ some people have said they are uncomfortable with the term “social prescribing” because it has perceived overtones of paternalism. Individuals may not appreciate being told what to do when it comes to their social sphere. However, as suggested by Malby and coauthors,^34^ social prescribing might be a useful label to adopt because the medicalized notion of prescribing encourages health care professionals and funders to take social prescribing schemes seriously and to view them as appropriate recipients of health care funding.

Even if the term “social prescribing” is deemed acceptable, the related jargon may be off‐putting: patients become “service users,” who “coproduce action plans” with “link workers” and “community sector organizations” to develop “holistic support networks” for their “nonclinical needs.” For people more comfortable and familiar with the idea of a doctor and a patient, such language might create confusion and reluctance to engage. The terminology may also lead to social prescribing being interpreted as stigmatizing and linked to statutory social services.^19^

Hence, careful thought needs to be given to how social prescribing and components within it are labeled and presented. There are likely to be trade‐offs between using descriptors that are sensitive and accurate, and those that are easily comprehensible to nonexperts. Use of accessible language could support “buy‐in” from both patients and health care professionals, an outcome that may be central to successful social prescribing.^2^

### Social Prescribing and the NHS System

#### Link Workers

The link worker's role in social prescribing is to help patients access potentially helpful community assets that offer opportunities to engage with others and receive assistance within the local community, often through activities and offerings run by voluntary organizations. This role requires link workers to be up to date on available community groups in a particular area. Link workers themselves may also deliver supportive interventions more directly: for instance, in their meetings with patients, they might engage in motivational interviewing or brief coaching.^35^

The link worker's role can call for significant emotional engagement, as they seek to present a particular kind of emotional persona that is consistent with the expectations of the organization (the NHS) and consumers (the patients). Engaging with people who have difficult or unfulfilling personal lives, and seeking to support them in finding solutions to their problems, can be highly demanding. If link workers are not provided with outlets for the challenges they experience as a result of the nature of their work, alongside training and techniques to cope with the emotional labor they undertake, then, as has been found for other caring professionals, they are likely to be vulnerable to workplace stress and burnout.^36^, ^37^ A survey of National Association of Link Workers members (n = 279) reported that nearly a third of the respondents were considering leaving their post in the next year, in part due to a lack of support or supervision.^38^

Uncertainty around the exact parameters of their role, and the absence of a well‐established history of the link worker profession (or associated qualification), could add to job‐related stress. Link workers are expected to perform a boundary‐spanning role. This means that they “facilitate transactions and the flow of information between people and groups who either have no physical or cognitive access to one another, or alternatively, who have no bases on which to trust each other.”^39^ In this role, they may help counteract the “tribalism” that can develop among health care providers (e.g., between health professionals and the VCS), acting as a go‐between for these different groups. However, link workers’ status and ability to succeed in their role may be hampered by the profession's lack of a recognized qualification and established history. This may be something that becomes less of a problem as time passes and new link workers are able to fit into a more established community of colleagues and learn from others’ experiences.

Some of these difficulties could be ameliorated if there were sufficient numbers of appropriately trained link workers to meet the demands of NHS needs. The current scheme plans on employing one link worker per primary care network. It remains to be seen if this arrangement will be adequate to ensure patients receive the support they need without overburdening link workers. Patient access to link workers is likely to be particularly important as we move forward from the COVID‐19 pandemic; however, these employees may be stretched beyond their professional capacity as the psychosocial and economic consequences of the pandemic surface.^38^

#### GPs

The success or failure of social prescribing will depend on the appropriate engagement and buy‐in from GPs,^2^ who tend to be responsible for referrals to link workers. GPs must therefore understand who is likely to benefit from social prescribing: patients who are able and willing to engage with the kinds of activities which link workers can direct them toward. There is some risk, as mentioned previously, that social prescribing could be seen as a way of off‐loading difficult patients who are not suitable for social prescribing, perhaps because of the complexity of their needs or the challenges of engaging with them.^35^ GPs’ efforts to refer difficult patients to link workers are not entirely unreasonable, as part of the rationale for introducing the social prescribing provision into primary care networks is to relieve GPs of some of their workloads.^11^ However, not all patients will be suitable for referral.

Redirecting patients to link workers could be an example of the diversification encountered within the primary care workforce as new roles are introduced and providers strive toward increasingly efficient allocation of work.^40^ In theory, this may result in a more cost‐effective way of outsourcing activities, freeing up GPs to concentrate on clinical cases. This is a type of task shifting, whereby less specialized members of staff are introduced “to address the shortage of human resources for health, improve access, save costs, and meet local needs.”^41^ However, when GPs are encouraged to focus narrowly on the patient's specific clinical problems, it is possible that the patient's physical situation will be divorced from any contributing psychosocial issues. For example, if a GP focuses narrowly on a patient's diabetes control, the physician might miss the contribution that noisy neighbors and not sleeping has on that person's well‐being and ability to engage in good self‐care. While strides have been taken to acknowledge and address the ways in which clinical needs are impacted by patients’ background circumstances (and social prescribing is a part of this), the carving up of the clinical and nonclinical realms could reinforce that distinction and dilute the quality of personalized care offered by the GP. Ideally, in the case of the patient with diabetes, the GP would still elicit information about broader contributing factors and then refer the patient to a link worker if the physician and other clinical staff lacked the detailed insight and capacity to address the patient's needs. Notably, this scenario requires that the GP broadly focus on nonclinical contributors to the patient's health before considering social prescribing for that patient.

One way to avoid care fragmentation is to ensure that there is an effective feedback system between link workers and GPs, so that the GP focuses primarily on the clinical needs of patients while nonetheless remaining alert to the ways in which other aspects of life may influence or affect each specific patient's ability to self‐manage their health and well‐being. Existing infrastructures (e.g., electronic health record systems) may facilitate communication between GPs and link workers. However, link workers would need permission to access patients’ data, and providing this access would involve both placing trust in link workers to keep records confidential and adapting the electronic health record system infrastructure. The British Medical Association has noted the importance of adding social prescribing as a default option for GP information technology systems.^42^

GPs may not understand the extent to which link workers require information about a patient's medical history or ongoing treatment. Ordinarily, such information would only be shared when clinically necessary for the care of the patient. However, when moving from clinical care within the NHS to well‐being promotion through structures external to the NHS, such information sharing should be given due consideration. One way to facilitate appropriate communication about patient care would be to develop platforms for members of the broader primary care team to meet in a multidisciplinary format to share knowledge and experiences. This still leaves the difficulty of determining what information ought to be shared (i.e., what is relevant to the link worker's capacity to perform their role), and how information sharing can be executed so it is consistent with the demands of consent procedures, ensuring that patients have sufficient understanding to make informed decisions about how their data are treated.^43^, ^44^

### Social Prescribing and Community Assets

#### Funding and Resources

For social prescribing to function effectively, the VCS must be able and willing to provide the kinds of support or engagement opportunities required. Although there are documented benefits to volunteering, and many volunteers may have specialist training and skills, reliance on voluntary services raises concerns that existing community assets may not be able to handle the increased demand without receiving additional resources or training.^45^, ^46^, ^47^, ^48^ Patients referred to VCS organizations via social prescribing could have increased vulnerability relative to those who normally engage with community groups. There is therefore a risk that community assets could become overwhelmed and struggle to cope, especially if they do not receive financial support or training. Expecting people to voluntarily provide services to assist the NHS without receiving additional support in return could be exploitative. Guidance from NHS England recommends that link workers gather information about the likely impact of social prescribing on VCS organizations, but it is unclear what influence this information will have on social prescribing delivery methods.^49^

The COVID‐19 pandemic has exacerbated the struggles of VCS organizations to meet social prescribing needs. Although the National Emergencies Trust has allocated £5 million funding for local groups responding to the COVID‐19 pandemic (e.g., food banks, groups supporting families and children),^50^ this funding is unlikely to mitigate completely the crisis facing the VCS as a consequence of the pandemic. Many organizations have faced budget shortfalls because fundraising events were cancelled and charity shops closed their doors, with attention redirected to dealing with the immediate physical impacts of the virus.^51^

If community assets are offered additional resources and training to assist with providing social prescribing activities, this could influence community groups’ behavior. For example, if there were additional money attached to VCS services for older people, community groups might focus more on provision for this demographic, perhaps at the cost to their youth services, and this shift in funding and service priorities could have benefits and harms. Instead of designating funding for specific populations or purposes, it might be more beneficial to ensure that funding provisions are sensitive to local needs. If incentives are misaligned, and service provision is poorly attuned to local needs (focusing instead on the most visible or sympathetic groups), or if evidence about effectiveness is lacking or misinterpreted, the outcome of social prescribing on community assets could be damaging.

#### Fitting Social Prescribing into a Health Care System

The formalization of social prescribing within the NHS (or alternative health care systems in other countries), and the administrative activities that this is likely to bring with it (e.g., additional checks and balances, paperwork, quality assessment of services), could endanger some of the existing advantages of VCS work, such as its flexibility, informality, and personal approach. Systems introduced to track good practice and health and safety are often experienced as controlling and misdirected by those forced to comply with them.^52^ Where such procedures and requirements were previously absent, VCS groups might be understandably reluctant to introduce them because they may place additional burdens on already limited resources.

The influence of health services’ use of VCS organizations to supply social prescribing activities could vary. For instance, groups that already provide such activities could be in higher demand and become better supported than less‐established groups. Similarly, groups able to adapt to the demands of working closely with the NHS (e.g., able to meet the increased bureaucratic burden and to form good relationships with link workers) may find their services expanding, whereas groups not in a position to do so may shrink. Because it will be difficult for link workers to remain aware of all available local community assets, there is a risk that they will rely on a limited set of groups/providers with which they are familiar instead of making referrals to community assets that might better serve patients but are unknown or untested by the link worker. Thus, one consideration in the rollout of social prescribing services will be the ways in which information about community assets is collected, updated, and made available to link workers. Platforms that provide searchable databases of social support programs and facilitate referrals, such as Aunt Bertha and NowPow in the United States, may help ensure that link workers do not overlook available resources, assuming such platforms are comprehensive and usable.^53^, ^54^

## Conclusion

There is enthusiasm to make use of social prescribing and to scale up its provision within the United Kingdom and elsewhere. Guidance from NHS England has been developed to assist and support GPs, link workers, VCS organizations, and others involved in planning and providing social prescribing services^49^; initiatives are also being developed in countries such as Canada, the United States, Australia, and New Zealand.^5^, ^6^, ^7^, ^8^, ^9^ Tangible benefits to patients and care providers are possible. However, despite policy‐driven enthusiasm, there are gaps in our knowledge of how social prescribing could and should be operationalized in real‐world settings. There remains significant variation in what social prescribing looks like, its target populations, and how it is provided. All the different actors, from those within health service networks to those external to them, need sufficient information and resources to be able to carry out their roles and work together productively. At the same time, more information is required regarding if and when social prescribing is effective at helping patients to self‐manage and support their nonclinical needs in ways that ultimately promote health and well‐being.

The issues we have discussed here relating to the role of patients, link workers, GPs, and community assets require further consideration and empirical research to better understand how they affect the potential for social prescribing to deliver its projected benefits, while avoiding unacceptable harms. Although we have highlighted areas of ethical significance and potential harm, it is also important to bear in mind that, if effective, social prescribing could be ethically required. That is, if social prescribing promotes health and well‐being in resource‐constrained environments, failing to establish social prescribing systems could have opportunity costs that are too great to justify.

1

*Funding/Support*: This research was funded in whole, or in part, by the Wellcome Trust [Grant number WT104848/Z/14/Z]. For the purpose of open access, the author has applied a CC BY public copyright licence to any Author Accepted Manuscript version arising from this submission.

*Conflicts of Interest Disclosure*: All authors completed the ICMJE Form for Disclosure of Potential Conflicts of Interest. No conflicts were reported.
